# A New Sandwich ELISA for Quantification of Thymidine Kinase 1 Protein Levels in Sera from Dogs with Different Malignancies Can Aid in Disease Management

**DOI:** 10.1371/journal.pone.0137871

**Published:** 2015-09-14

**Authors:** Kiran Kumar Jagarlamudi, Laura Moreau, Sara Westberg, Henrik Rönnberg, Staffan Eriksson

**Affiliations:** 1 Department of Anatomy, Physiology, and Biochemistry, Veterinary Medicine and Animal Science center, Swedish University of Agricultural Sciences, Uppsala, Sweden; 2 University Animal Hospital, Swedish University of Agricultural Sciences, Uppsala, Sweden; 3 Center of Clinical Comparative Oncology (C_3_O), Department of Clinical Sciences, Swedish University of Agricultural Sciences, Uppsala, Sweden; Hunter College of The City University of New York, UNITED STATES

## Abstract

Thymidine kinase 1 (TK1) is a DNA precursor enzyme whose expression is closely correlated with cell proliferation and cell turnover. Sensitive serum TK1 activity assays have been used for monitoring and prognosis of hematological malignancies in both humans and dogs. Here we describe the development of a specific sandwich TK1-ELISA for the quantification of TK1 protein levels in sera from dogs with different malignancies. A combination of rabbit polyclonal anti-dog TK1 antibody and a mouse monoclonal anti-human TK1 antibody was used. Different concentrations of recombinant canine TK1 was used as standard. Clinical evaluation of the ELISA was done by using sera from 42 healthy dogs, 43 dogs with hematological tumors and 55 with solid tumors. An established [^3^H]-dThd phosphorylation assay was used to determine the TK1 activity levels in the same sera. The mean TK1 activities in dogs with hematological tumors were significantly higher than those found in healthy dogs. In agreement with earlier studies, no significant difference was observed in serum TK1 activities between healthy dogs and dogs with solid tumors. However, the mean TK1 protein levels determined by new TK1-ELISA were significantly higher not only in hematological tumors but also in solid tumors compared to healthy dogs (mean ± SD = 1.30 ± 1.16, 0.67 ± 0.55 and 0.27± 0.10 ng/mL, respectively). Moreover, TK1-ELISA had significantly higher ability to distinguish lymphoma cases from healthy based on receiver operating characteristic analyses (area under the curve, AUC, of 0.96) to that of the activity assay (AUC, 0.84). Furthermore, fluctuations in TK1 protein levels during the course of chemotherapy in dogs with lymphoma closely associated with clinical outcome. Overall, the TK1-ELISA showed significant linear correlation with the TK1 activity assay (*r*_*s*_ = 0.6, p<0.0001). Thus, the new TK1-ELISA has sufficient sensitivity and specificity for routine clinical use in veterinary oncology.

## Introduction

Dogs are frequently affected with various neoplastic diseases such as lymphomas, leukemias and mammary tumors. Lymphomas are the most common form of hematological tumors and account for 5% of all canine cancers. The annual incidence has been estimated to 13 to 40 cases per 100,000 dogs [1, 2]. Canine lymphoma is similar to human non-Hodgkin’s lymphoma in terms of genetic and environmental factors that contribute to disease progression [3, 4]. Early stage diagnoses in combination with effective chemotherapy increase the life span of the affected dogs. Proliferation markers are valuable in order to detect tumor diseases at an early stage, as uncontrolled cell proliferation is one of the hallmarks of cancer. Several proliferation markers including argyrophilic nucleolar organizing regions (AgNORs) [5], proliferation cell nuclear antigen (PCNA) [6] and Ki-67 [7] have been investigated as prognostic markers in canine lymphoma, but their usage is limited to immunohistochemistry. Serum lactate dehydrogenase (LDH) was also investigated as a marker for monitoring of canine lymphomas, but LDH is up regulated in diseases other than malignancies and thus has a limited clinical value. Furthermore, serum LDH levels did not correlate to either stage or prognosis in a large study on canine lymphoma [8, 9, 10].

Thymidine kinase 1 (TK1) is one of the biomarker that is released into the blood during uncontrolled cell proliferation [11]. TK1 converts deoxythymidine (dT) to deoxythymidine monophosphate (dTMP), which eventually incorporated into the DNA strand [12]. Further, TK1 activity is tightly associated with different phases of cell cycle and it reaches a peak in S-phase, declines rapidly in G_2_, and is degraded by specific mechanism in M phase [13, 14, 15].

Serum TK1 activity measurement is an established tool for diagnosis and monitoring of lymphomas and leukemias in human medicine [16, 17, 18]. Serum TK1 activity is measured by using several enzymatic assays e.g. the TK-REA or TK-Liaison assays. Studies have shown that both the TK-REA and the TK-Liaison assays provide valuable information for prognosis and treatment monitoring of canine hematological tumors [9, 19]. Recent study, using the natural substrate [^3^H]-dT (deoxythymidine) instead of substrate analogs showed that this assay was equally sensitive to TK-REA and TK-Liaison assays. This assay can also be used for diagnosis and treatment monitoring of canine lymphomas [20]. However, TK1 activity assays could not differentiate sera from canine solid tumors compared to healthy controls [9, 20]. This limits the clinical implications of TK1 activity assays in canine oncology.

The development of antibodies against different regions of human TK1 has suggested an alternative way of TK1 determinations [21, 22]. Several clinical studies showed significantly higher TK1 protein levels in sera from patients with solid tumors compared to healthy persons [23, 24]. TK1 protein assays were found to be more sensitive than the TK1 activity assays for prognosis and treatment monitoring of patients with solid tumors [25, 26, 27, 28].

The crystal structure of human TK1 has been resolved [29] and most of the enzyme, including the N-terminal region remains conserved in humans and dogs but a significant difference (9.1%) is found in the C-terminal region [12, 30]. Recently, we established an Immunoaffinity assay which is based on antibodies against different peptides from C-terminal region of dog TK1 [31]. This allowed determination of serum TK1 protein levels in dogs with various malignancies and the TK1 protein assay could differentiate dogs with solid tumors from healthy dogs [31]. In a subsequent study, the TK1 protein levels were found to be significantly higher in sera from dogs with mammary adenomas compared to healthy dogs, whereas this was not observed when the TK1 activity levels were measured [32]. The aim of our work was to translate the results from previous studies into a sensitive and reproducible TK1 protein assay format for detection and quantification of TK1 in sera from dogs with malignancies. The goal was achieved by establishing a sandwich ELISA which widens the utility of TK1 in routine clinical practice. The development of such an assay is described as well as determinations of the TK1 protein levels and the TK1 activities in dog serum samples. Furthermore, serum samples from 7 dogs with lymphoma were followed during therapy with the TK1 activity and protein assays.

## Materials and Methods

### Serum samples

Serum samples from healthy dogs, dogs with hematological tumors and solid tumors were collected at the University Animal Hospital at the Swedish University of Agricultural Sciences, Uppsala, Sweden, and stored at -20°C until analysis. Informed consent was obtained from all owners to use their dogs in this study. The project was approved by the Swedish Animal Ethics Committee (ref no. C12/15). The tumor diagnostic procedures were as described previously [10]. The study comprised samples from healthy dogs (n = 42), dogs with hematological tumors (n = 43: lymphoma n = 38, leukemia n = 5) and dogs with solid tumors (n = 55; 22 mammary tumors, 12 malignant melanomas, 10 mastocytomas and 11 other tumors). The mean and median age was 6 years (range 2–10 years) for the healthy group, 8.5 and 8 years (range 3–13 years) for hematological tumors, and 8.5 and 8 years (range 1–14 years) for dogs with solid tumors. Further information about dog breed, age and sex are described in supporting information (Table A, Table B and Table C in S1 File). Five Dogs with malignant lymphoma were treated with a doxorubicin-based multi agent protocol which consisted of doxorubicin, L-asparaginase, cyclophosphamide, chlorambucil, hydroxyurea, and prednisone [33] and serum samples were taken from the same individual along the course of chemotherapy. Two dogs were treated with a standard cyclophosphamide, vincristine (oncovin), and prednisolone (COP) protocol as described previously [34].

#### TK1 antibodies and recombinant dog TK1

**Fig 1:**
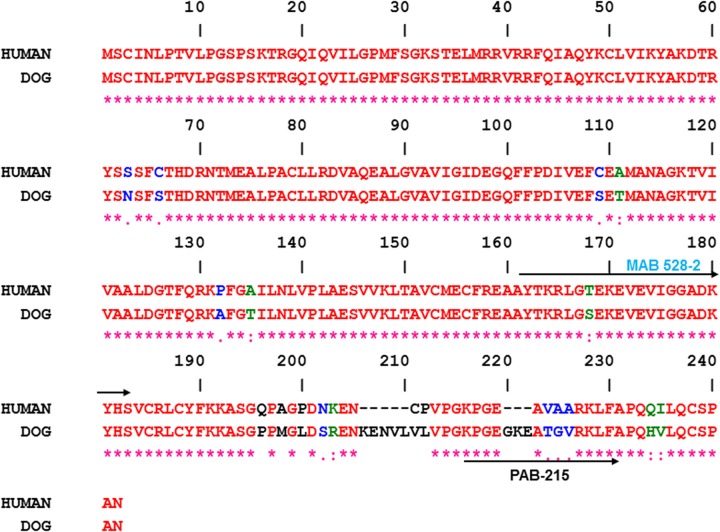
Amino acid sequence alignment of human and dog TK1. Residues that differ between the two sequences are shown in different colour. Antibodies raised against two different regions of TK1 sequence are indicated by arrows; mouse monoclonal antibodies MAB 528–2 (161–183 AA) and rabbit polyclonal antibodies PAB-215 (215–231 AA). Gene Bank accession numbers are XM_540461 (dog) and KO_2582 (human).

A polyclonal anti-dog TK1 antibody was raised against the 16-amino acid synthetic peptide from the C-terminal region of dog TK1 (amino acids 215–231 KPGEGKEATGVRKLF; PAB-215) (Fig 1) in rabbits (GenScript, Piscataway, NJ, USA). The antisera were collected after the 3^rd^ and 4^th^ immunizations and purified on peptide coupled Sepharose 4B columns as described [22]. Mouse monoclonal anti-human TK1 antibody was produced against the long lasso shaped loop of human TK1 (amino acids 161–183, AYTKRLGTEKEVEVIGGADKYHS; MAB 528–2) as described previously [35]. For both antibodies, a cysteine was added to the N-terminus and used for coupling to carries protein. The MAB 528–2 antibodies were provided by AroCell AB, Uppsala, Sweden. The recombinant dog TK1 was cloned and expressed in *E*.*Coli* and purified by Ni- Sepherose affinity chromatography as previously described [30].

#### Biotinylation of MAB 528–2

The MAB 528–2 antibodies were biotinylated using the ChromaLink™ Biotin Antibody Labeling Kit (Solulink, California, USA) according to manufacturer’s instructions. Biotinylation was checked by Western blotting using streptavidin-HRP.

### The serum TK1 activity assay

Thymidine kinase 1 activity in serum samples was determined using the optimized [^3^H]-dThd phosphorylation assay, as described previously [20, 31]. In brief, 10 μL of serum was incubated with the reaction mixture containing 10 mM Tris-HCl pH 7.6, 2 mM Dithiothreitol (DTT), 5 mM MgCl_2_, 5 mM NaF, 5 mM ATP, and 5 μM [^3^H]- deoxy thymidine for 1 hr at 37°C. Aliquots were then applied to the DE-81 filter paper discs, which were washed twice with 1 mM ammonium formate for 5 min/each. The bound products were eluted for 45 min in 0.1 M HCl and 0.2 M KCl and the radioactivity was determined. The TK1 activity was expressed as pmol/min/mL.

### Sandwich TK1-ELISA

ELISA plates of the type Maxisorp, NUNC (Denmark) were coated with 100 μL/well of the PAB-215 antibody diluted to a final concentration of 4 μg/ml in 100 mM carbonate buffer (pH 9.6). The coated plates were incubated at 4°C overnight then washed four times with wash buffer (Tris 0.1 M, pH 7.4, NaCl 0.3 M, Tween 0.05% and BSA 1%). Thereafter, all wells were blocked with 200 μL/well of 5% nonfat dry milk in TBS and 0.1% Tween 20 (TBS-T) for 1 hr at room temperature. Dog sera of 50 μL were diluted in 50 μL serum dilution buffer incubated at room temperature for 1 hr. Recombinant dog TK1 was diluted serially to generate a standard curve with concentrations ranging from 0.6 to 10 ng/mL. Wells were washed and diluted recombinant dog TK1 and serum samples were added. After 2 hr incubation at room temperature on a rocking platform, plates were washed again and 100μL/well of biotinylated MAB528-2 antibody (4 μg/mL in wash buffer) was added followed by incubation with shaking for 1hr. The plates were washed as above and streptavidin-horse radish peroxidase (HRP) diluted 1: 32,000 in wash buffer was added, and incubated for 1 h. After the final wash, 3, 3 _, 5, 5 _-Tetramethylbenzidine (TMB) (Thermo Fisher Scientific) was added and allowed to develop colour for 15 min, and then quenched with 2N H_2_SO_4_. The O.D values in wells were read at 450 nM in an Infinite M200 Micro plate reader (Tecan, Mannedörf, Germany).

### Validation of the dog TK1 ELISA

Dog recombinant TK1 was purified as described previously [30] and a standard curve established with different concentrations of recombinant dog TK1 (0.6–10 ng /mL diluted in sample dilution buffer). By using the standard curve, the average absorbances of different serum samples in duplicate were used to determine the concentrations of TK1 in the serum samples. The inter assay variation was determined from the mean and SD of all serum samples which were independently assayed as duplicates in two different experiments.

### Statistical analysis

The TK1 activity and TK1 protein levels in sera from healthy dogs, and dogs with hematological tumors and different solid tumors were tested for normal distribution by using the D’Agostino and Pearson omnibus normality test. Both TK1 activity and protein levels in healthy, dogs with hematological tumors as well as with solid tumors showed non-Gaussian distribution. Spearman correlation coefficients (*rs)* were used to determine the correlation between TK1 activities and TK1 protein levels. STK1 activity and STK1 protein levels in the different groups were log transformed (natural log) before analysis. The Mann Whitney t-test was used to evaluate the difference between the groups. Kruskal-Wallis tests followed by Dunn’s Multiple Comparison post-tests were used to compare TK1 activity and TK1 protein across multiple groups. For sensitivity and specificity analysis and for comparison of the two assays, receiver operating characteristic (ROC) curves were constructed. Statistical analyses were performed using Graph Pad Prism 5.0 (Graph Pad Software, La Jolla, CA, USA). The level of significance was set at P<0.05.

## Results

### Description of the ELISA procedure

**Fig 2:**
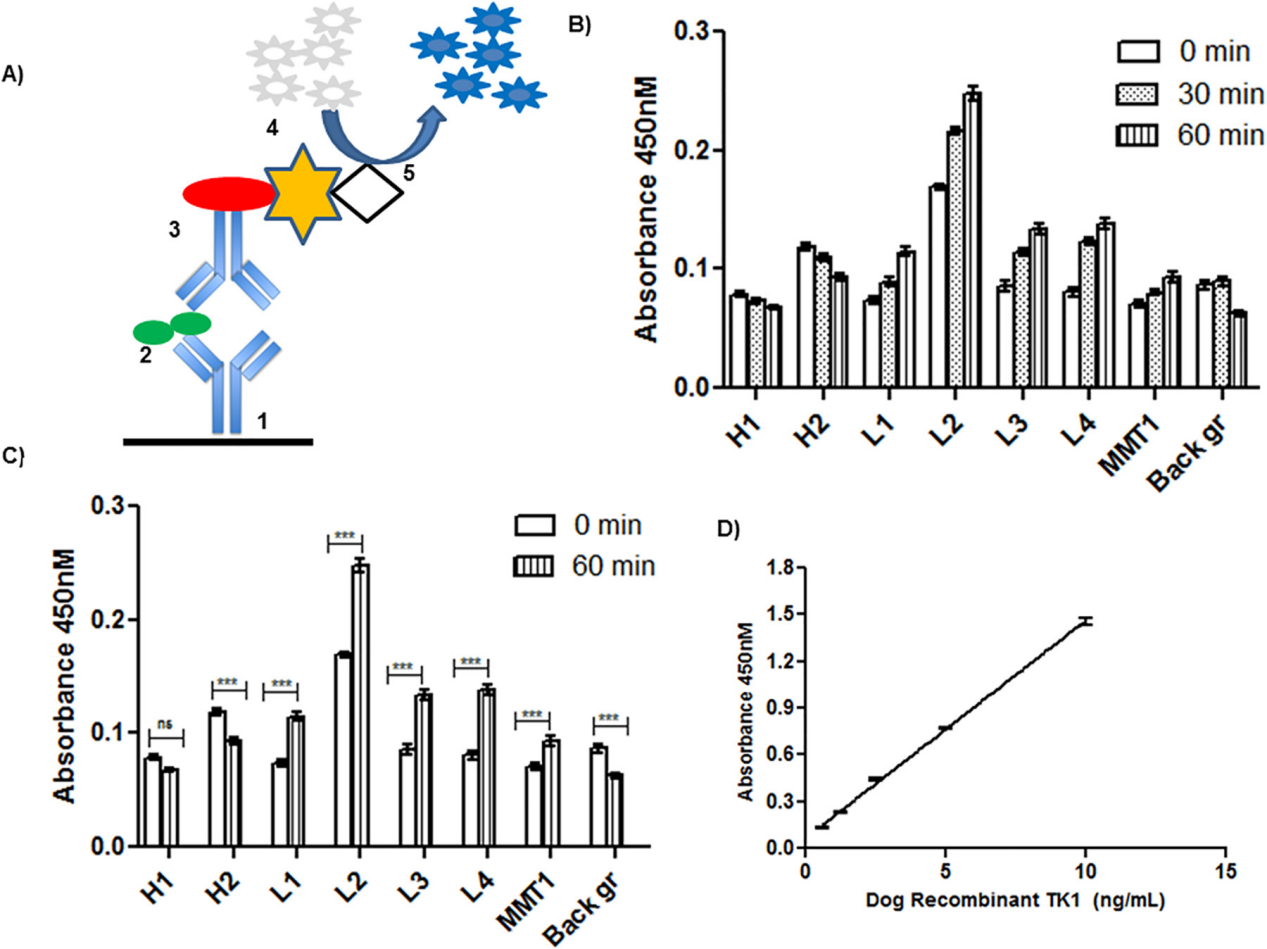
ELISA procedure, effect of buffer during pre-incubation and standard curve. (A) Schematic presentation of the sandwich ELISA. The five steps are: (1) coating of microplate wells with polyclonal dog anti-TK1 antibody (C215-231), (2) binding of TK1 in serum to coated antibody, (3) attachment of the biotinylated MAB 528–2 to TK1 in serum, (4) detection of biotin by streptavidin-HRP, and finally (5) enzymatic activity monitored by addition of 3,3 _, 5,5 _-Tetramethylbenzidine (TMB) chromogenic substrate. (B) Absorbance values of healthy, lymphoma and mammary tumor sera on TK1-ELISA during different pre-incubation time points (0, 30 and 60 min). The error bars represents the SEM of the mean values. (C) Comparison of the absorbance values of the serum samples from 0 min and 60 min. The error bars represent SEM of the mean. ns = no significant difference, *** *p* < 0.001. (D) Standard curve with different concentrations ranging from 0.6 to 10 ng/mL of recombinant dog TK1 plotted against the absorbance at 450 nM from 10 individual runs. The error bars represents the SEM of the mean values.

The ELISA principle is based on a sandwich immunoenzymatic system as shown in Fig 2A. The first step is coating of micro titer plates with purified polyclonal anti dog TK1 specific antibody produced against a peptide in the C-terminal region of dog TK1 (PAB-215, Fig 1). This part of TK 1 is an exposed region of the TK1 protein complexes formed in the blood as shown by earlier work with human and dog serum TK1 [15, 30]. After blocking the wells with milk powder (5%), pre-incubated serum samples from healthy and dogs with malignancies were allowed to bind to the antibodies on the plates. Proteins not specifically bound are removed by the washing procedure. The second antibody, MAB 528–2 was produced against the highly conserved and exposed active site region of TK1 [35]. This antibody was biotinylated and allowed to react with the TK1 bound to the wells. The detection of the antigen-antibody bound complex by a streptavidin-peroxidase (HRP) complex was visualized by the addition of a chromogenic substrate (TMB). The intensity of the colour reaction is proportional to the quantity of TK1 present in the serum samples.

The serum samples were pre-incubated with a special serum dilution buffer that allows modifications in the TK1 protein to increase the specificity of the antibody reaction. Some studies were done to determine the effects of the pre-incubation step by incubating serum samples from different tumors. Serum samples were pre-incubated with the serum dilution buffers for 0, 30 and 60 min (Fig 2B). Pre-incubation for 1 hour with serum dilution buffer led to a significant reduction in the absorbance of sera from healthy dogs. Simultaneously, there was a 5–20% increase in the absorbance observed with sera from dogs with tumors (Fig 2C). These results suggested that the pre-incubation procedure leads to modification in the serum TK1 complexes, so that it can react with the antibodies more efficiently. Therefore, the pre-incubation step gave increased sensitivity and reproducibility of the canine TK1-ELISA.

Dog recombinant TK1 was used as a calibrator to generate a dose-response curve for the TK1- ELISA. A typical calibration curve is shown in Fig 2D. Intra-assay variation (CVs) at all non-zero calibration points was ≤ 10% and between-run imprecision was <15% at concentrations down to 0.6 ng/mL. For serum samples the inter-assay variation ranges from 5–15% and intra assay variation is 5%. The mean serum TK1 activities and TK1 protein levels in healthy dogs, dogs with hematological malignancies and with solid tumors are shown in supporting information (S1 File) as described separately below.

#### STK1 activity levels in healthy dogs and dogs with hematological tumors

**Fig 3:**
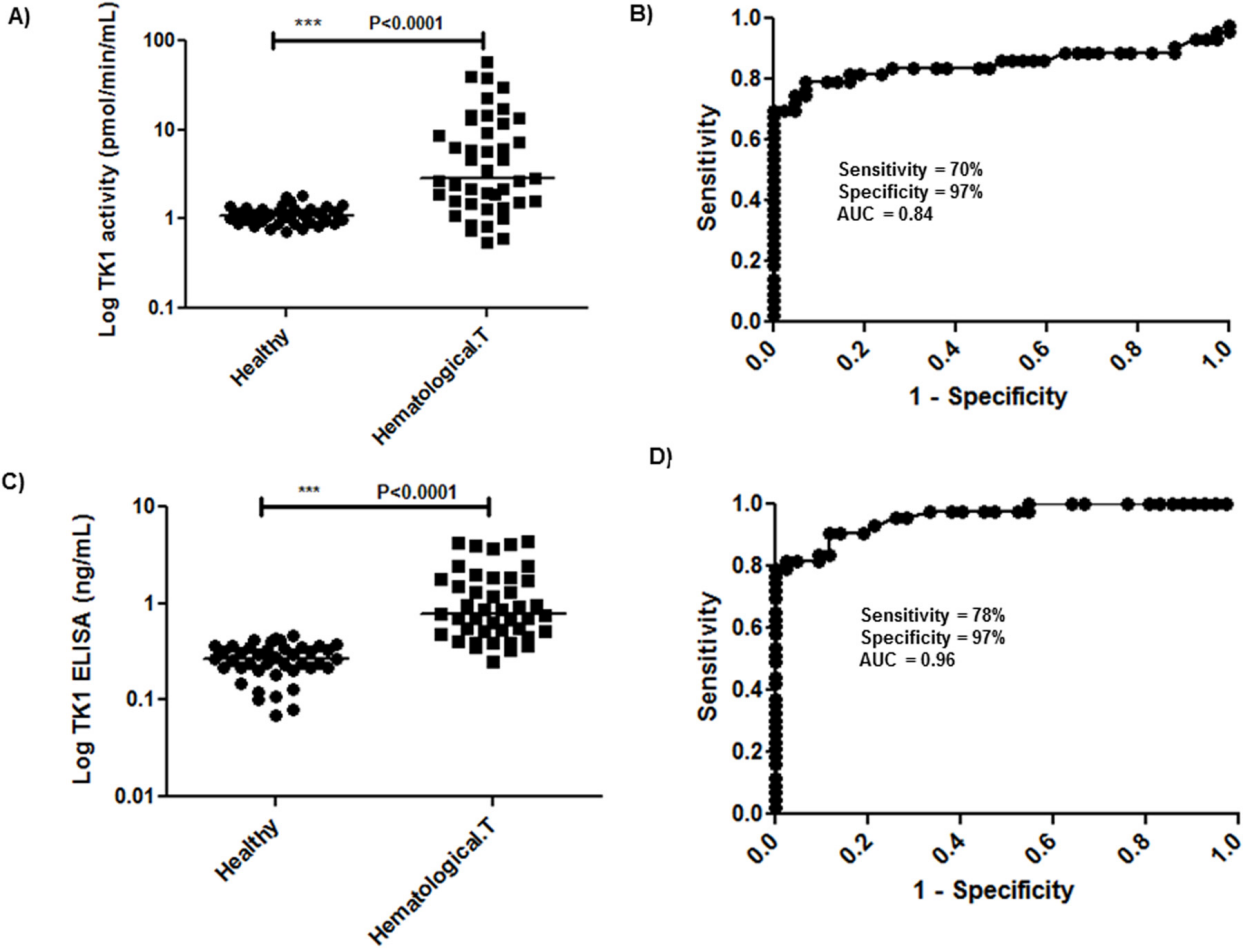
Log serum TK1 activity and protein determinations in healthy dogs and dogs with hematologic malignancies. (A) STK1 activity distribution (pmol/min/mL) in sera from healthy dogs and dogs with hematological tumors.The error bars represent median. (B) Receiver operating characteristic (ROC) curve of STK1 activity assay for discrimination of hematological tumor dogs (n = 43) from healthy (n = 42). (C) STK1 protein levels in sera from healthy dogs and dogs with hematological tumors. The error bars represent median. (D) ROC curve analysis for the STK1 protein levels of healthy in comparison to those in dogs with hematological tumors.

In the healthy dogs, STK1 activities were in the range of 0.7–1.8 pmol/min/mL with a mean and standard deviation (SD) of 1.12 ± 0.25 pmol/min/mL. The STK1 activity levels in sera from dogs with hematological tumors were 0.5–60 pmol/min/mL (mean ± SD = 8.6 ± 12.3). In healthy dogs as well as in dogs with hematological tumors, no significant difference was found in STK1 activity levels between males and females. Statistical analysis using the Mann–Whitney U test showed that STK1 activity was significantly higher in the hematologic tumor group compared to the healthy group (P<0.0001, Fig 3A). The ROC curve analysis of the STK1 activity assay had an area under curve (AUC) of 0.85 (P<0.0001, 95% CI, 0.75–0.94) (Fig 3B). At the cut-off value of 1.8 pmol/min/mL, the sensitivity was 70% (95% CI, 0.54–0.83) and the specificity 97% (95% CI, 0.87–0.99).

#### STK1 ELISA determinations in healthy dogs and dogs with hematological malignancies

TK1 protein levels in clinical samples were determined by using different concentrations of recombinant dog TK1 as calibrator. In healthy dogs STK1 protein levels are in the range of 0.07–0.47 ng/mL (mean ± SD = 0.27 ± 0.1). The estimated upper limit of the normal reference based on 42 healthy dogs was 0.47 ng/mL (mean ± 2SD). Sera from dogs with hematological malignancies had higher STK1 protein levels, ranging from 0.25 to 4.4 ng/mL (mean ± SD = 1.30 ± 1.16). Significant differences were found in the mean STK1 protein levels between healthy dogs and dogs with hematological tumors (P<0.0001, Fig 3C). The ROC curve analysis showed an AUC of 0.96 (P< 0.0001, 95% CI, 0.92–0.99), and the sensitivity was 78% (95% CI, 0.64–0.89) and the specificity was 97% (95% CI, 0.87–0.99), using a cutoff value of 0.47 ng/mL (Fig 3D).

#### STK1 activity and STK1 protein levels in sera from dogs with solid tumors

**Fig 4:**
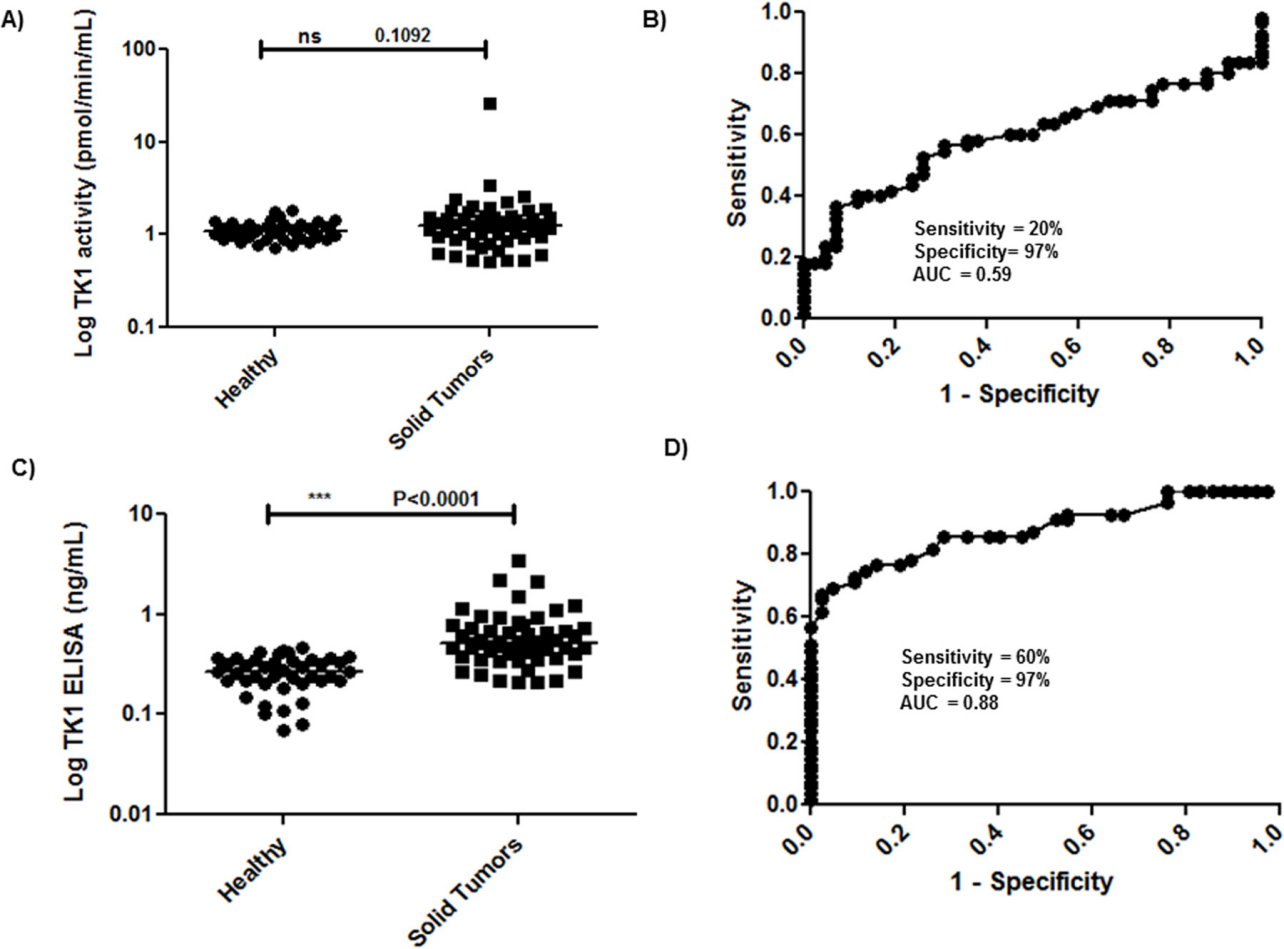
Log serum TK1 activity and protein determinations in healthy dogs and dogs with solid tumors. (A) STK1 activity distribution (pmol/min/mL) in sera from healthy dogs and dogs with solid tumors.The error bars represent median. (B) Receiver operating characteristic (ROC) curve of STK1 activity assay for discrimination of solid tumor dogs (n = 55) from healthy (n = 42). (C) STK1 protein levels in sera from healthy dogs and dogs with solid tumors. The error bars represent median. (D) ROC curve analysis for the STK1 protein levels to distinguish healthy from dogs with solid tumors.

Most of the sera from dogs with solid tumors had STK1 activity below the cut-off value and the STK1 activity levels ranged from 0.5–26 pmol/min/mL (mean ± SD = 1.7 ± 3.3). There was no significant difference (P = 0.1092) between the values found in dogs with different forms of solid tumors and healthy dogs (Fig 4A). These results are quite similar to the previous studies [11, 20]. The ROC curve analysis showed an AUC of 0.60 (P = 0.108, 95% CI, 0.48–0.71), the sensitivity was 20% (95% CI, 0.09–0.31) with a specificity of 97% (95% CI, 0.87–0.99), using a cutoff value of 1.8 pmol/min/mL (Fig 4B). However, the TK1 protein levels were significantly higher in sera from dogs with solid tumors (mean ± SD = 0.67 ± 0.55) compared to healthy (P<0.0001, Fig 4C). Furthermore, the ROC curve analysis had an AUC of 0.88 (P< 0.0001, 95% CI, 0.80–0.95) (Fig 4D). Using a cutoff value of 0.47 ng/mL, the sensitivity was 60% (95% CI, 0.47–0.74) and the specificity 97% (95% CI, 0.87–0.99). These results demonstrate that the TK1-ELISA could differentiate dogs with solid tumors efficiently from healthy dogs while the TK1 activity assay could not.

**Fig 5:**
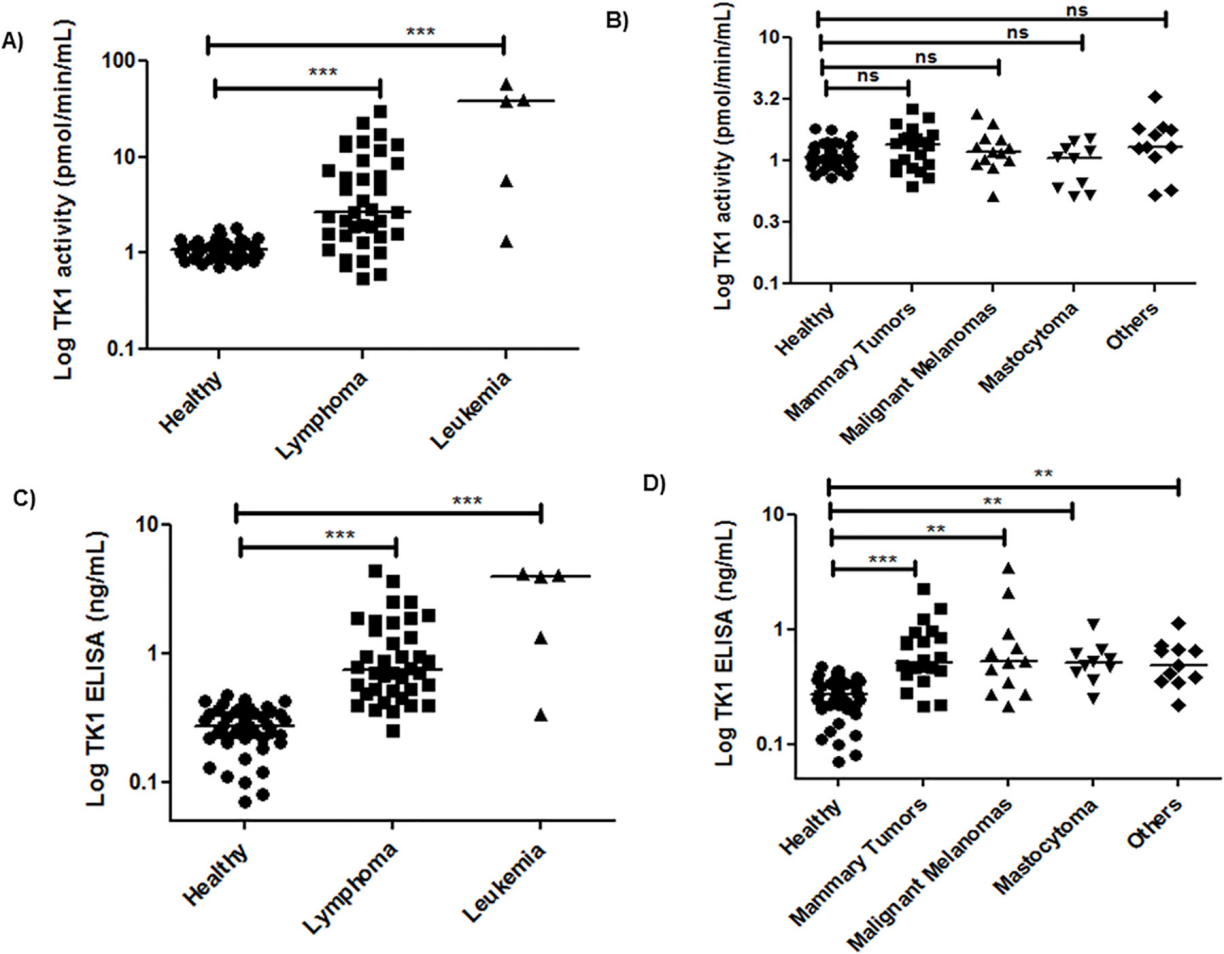
Log STK1 distribution in different sub groups of dog tumors. (A) STK1 activity levels (pmol/min/mL) in sera from healthy dogs, dogs with lymphoma, leukemia. (B) Log STK1 activity levels in healthy dogs, dogs with mammary tumors, malignant melanomas, mastocytomas and other tumors. The error bars represent median. (C) STK1 protein distribution in sera from healthy dogs, dogs with lymphoma, leukemia. (D) STK1 activity levels in healthy dogs, dogs with mammary tumors, malignant melanomas, mastocytomas and other tumors. The error bars represent median. * *p* < 0.05; ** *p* < 0.01; *** *p* < 0.001.

The Tumor groups were further subdivided into lymphomas, leukemias, mammary tumors, mastocytomas, malignant melanomas and some other tumor types as shown in Fig 5. The STK1 activity levels in these tumor groups were compared using the cut-off values based on TK1 activity in healthy sera. Most of the sera from lymphoma and leukemia patients were clearly above the cut-off (Fig 5A) (25/38 and 4/5, respectively). However, in case of the solid tumor sub groups only a minor part had activities above the cut-off, i.e. in case of mammary tumors (5/22), malignant melanomas (2/12) and other tumors (3/11) (Fig 5B). However, STK1 protein levels determined by the TK-ELISA in the different subgroups showed two different patterns i.e. in the lymphomas and leukemias most of the sera had STK1 protein levels above the cut-off (Fig 5C) (i.e. 30/38 and 4/5, respectively) similar to what was found with the STK1 activity. However, in case of the solid tumor groups STK1 protein levels were above the cut-off, i.e. in mammary tumors (14/22), malignant melanomas (7/12), mastocytomas (6/10) and other tumors (6/11) (Fig 5D). Whereas the STK1 activity assay values in healthy, hematological and solid tumor dogs were compared to TK1 ELISA values (ng/mL), a significant correlation between the two assays (r = 0.6, P<0.0001) was found, using linear regression analysis. Further correlations between two assays in each group along with their median values are shown in Table 1.

**Table 1:** STK1 activity and TK1-ELISA values in different groups and correlations between assays.

Group	STK1 activity (pmol/min/mL)		STK1-ELISA (ng/mL)		Correlation b/n STK1 activity &	*P value*
	Range	Median	Range	Median	STK1-ELISA(*rs*)	
Healthy (n = 42)	0.73–1.82	1.09	0.07–0.47	0.27	0.49	0.001
Hematological.T (n = 43)	0.56–58.7	2.87	0.25–4.38	0.78	0.77	<0.0001
Solid Tumors (n = 55)	0.51–25.8	1.25	0.21–3.4	0.51	0.36	0.0098

***rs*** = Spearrman correlation co-efficient

### Serum TK1 activity and protein levels followed during chemotherapy of dogs with lymphoma

**Fig 6:**
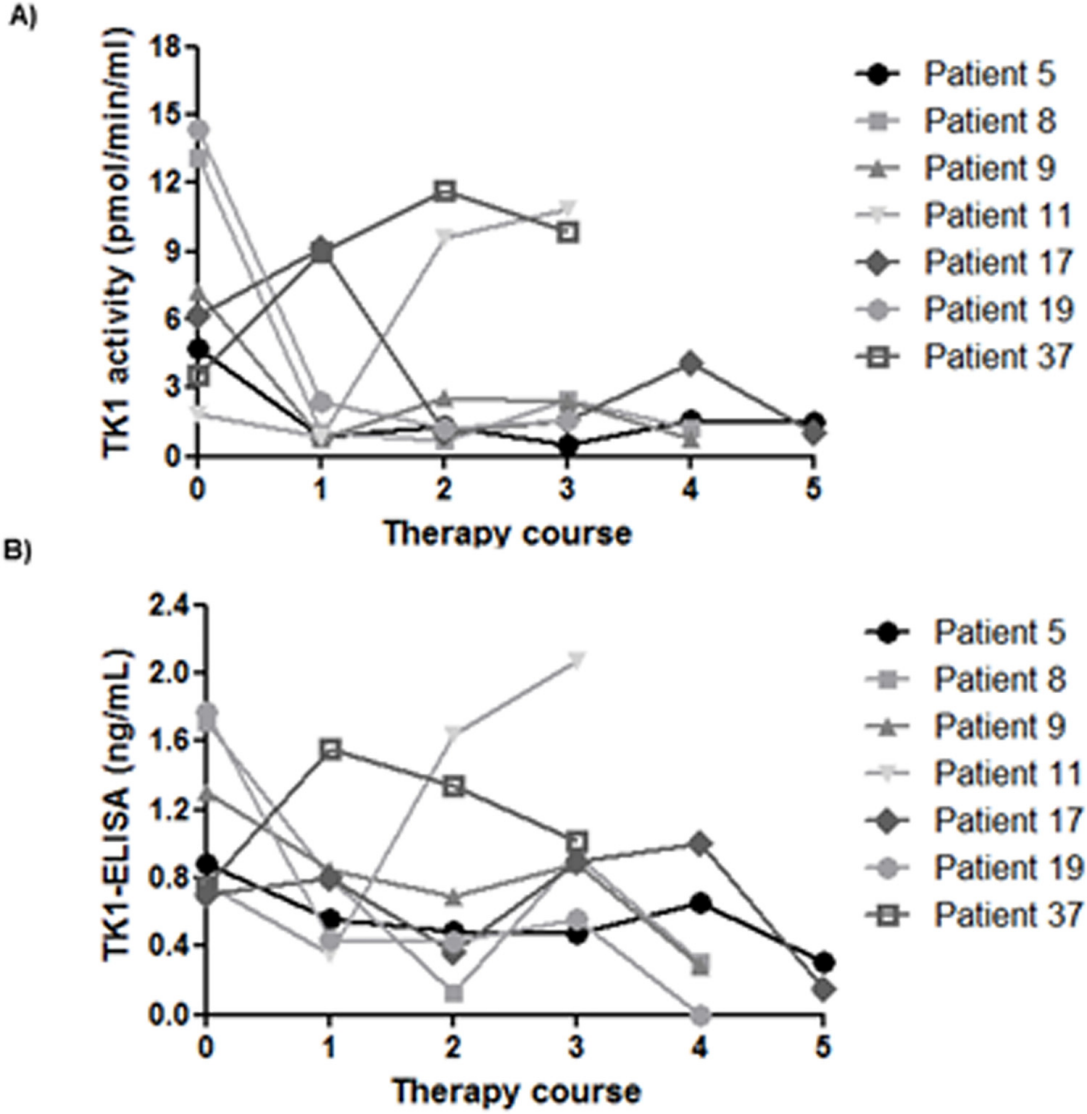
Serum TK1 activity and protein profiles during chemotherapy of dogs with lymphoma. (A) STK1 activity levels (B) STK1 protein levels in sera from dogs with lymphoma during the course of therapy.

7 out of 38 lymphoma patients were further followed during the course of therapy. Dogs with lymphoma are often treated with a doxorubicin based multi-agent protocol (patient’s no. 5, 8, 9, 11, 17) (Table B in S1 File). Two dogs were treated with COP protocol (Patient no. 19, 37). Serum samples were collected from dogs at the time of diagnosis and after each dose of ADRIA-plus. STK1 activity and STK1 protein levels were determined by using dThd assay and TK1-ELISA. Initially, high STK1 activity and STK1 protein values were found in six dogs, and five dogs showed significant decline in STK1 activity and STK1 protein levels after the first treatment, leading to levels similar or lower than the cutoff value (Fig 6A and 6B). In two patients (no. 8, 9) there was an increase in STK1 activity and TK1 protein during the 3^rd^ treatment, but both the patients appeared to be in remission after the 4^th^ treatment (Fig 6A and 6B). In patient no. 5 and 17, STK1 activity and STK1 protein levels increased after the 1^st^ and decreases after 2^nd^ treatment, but increased after the 3^rd^ and 4^th^ treatment and then decreased after the 5^th^ treatment (Fig 6A and 6B). Patient no. 19 was apparently in complete remission after the 2^nd^ treatment. Mean serum TK1 activity and TK1 protein in dogs with lymphoma that were in complete remission (CR) were not significantly different from the TK1 in healthy controls (*P* = 0.342; *P* = 0.417). However, two patients (no: 11, 37) had shown a different pattern of response to chemotherapy. Patient 11 had significant reduction in TK1 activity and TK1 protein levels after 1^st^ treatment. Thereafter, TK1 levels were increased after 2^nd^ and 3^rd^ treatment and this patient had tumor relapse which did not respond further to the treatment. Whereas, patient 37 had significant increase in TK1 activity and TK1 protein levels after 1^st^ treatment. During the further course of therapy, a slight decrease was found in TK1 levels after 2^nd^ and 3^rd^ treatments, still both the TK1 activity and TK1 protein levels were significantly higher compared to controls. Furthermore, this dog also did not respond to the treatment and both the dogs were euthanized later.

## Discussion

Previous studies have shown that serum TK1 activity [11, 19, 20] measurements are valuable for prognosis and treatment monitoring of canine hematological tumors. The results presented here support these conclusions. However, the activity assays are complex because they involve use of hazardous and expensive radioisotopes, thus limiting their clinical value. Since the ELISA format is commonly used in almost all clinical laboratories, developing such immunoassays would significantly increase the clinical accessibility and utility of TK1 as biomarker. In human medicine, studies with immunoassays based on TK1 antibodies have provided to be sensitive tools for prognosis and monitoring of different tumors [23, 28]. There have been several attempts to establish antibody based TK1-ELISA in human medicine but recently has a robust ELISA been available for research purposes only (AroCell AB, Sweden). This is probably due to the difficulty of obtaining antibodies with sufficient sensitivity and specificity to detect the serum forms of TK1. The complexity of TK1 oligomers found in serum, preventing reproducible reactivity with different type of anti TK1 antibodies, is most likely also a contributing factor. Pre-incubation with serum dilution buffer is one of the key factors that reduced the higher complex form of serum TK1 to lower forms. This contributes further not only to higher sensitivity of ELISA but also to reproducibility. Some attempts have been made to use antibody assays to detect and characterize serum TK1 in canine tumors. In one preliminary study, an antibody directed against the long lasso loop (AA 161–183) in the active site of human TK1 was used. It was clear that these antibodies detected cellular TK1 in canine lymphoma tissues [15]. In another study, recombinant, cellular and serum forms of the canine TK1 could be detected and characterized by using two antibodies raised against peptides from the C-terminal region of dog TK1. There are several similarities but also differences in the oligomeric structure of human and dog serum TK1 [30]. Furthermore, studies have shown that sera from dogs with solid tumors had low TK1 activity and high levels of TK1 protein compared to healthy dogs [31, 32].

Based on these previous studies, we developed a canine TK1-ELISA, which can overcome the limitations of traditional TK1 radioisotope assays. The ROC curve analysis of results with hematological tumors demonstrated that both the TK1-ELISA and TK1 activity assays have sensitivity more than 65% and a false positive rate of 3%. These results suggest that both the assays are sensitive enough for clinical routine practice for dogs with hematologic malignancies.

The most important finding in this study was that when sera from dogs with mammary tumors, melanomas, and mastocytomas were analyzed with the two TK1 assays, we observed that only 10 out of 55 dogs showed increased TK1 activity levels compared to healthy dogs. In contrast when the new TK1-ELISA was used to determine the levels in these subgroups, 33 out 55 sera had increased TK1 protein levels compared to those in healthy dogs. The ROC curve analysis of TK1-ELISA results showed a sensitivity of 60% compared to 20% with the TK1 activity assay and thus the TK1 ELISA appears to be suitable for clinical applications in case of dogs with solid tumor diseases. The reason for this is most likely related to differences in specific activity of serum TK1 since previous size exclusion analysis showed that serum TK1 exist as enzymatically active high molecular weight aggregates in dogs with lymphoma [30]. However, in sera from dogs with mammary tumor only a small portion of active high molecular weight TK1 aggregates were found and a large fraction of apparently inactive TK1 protein [32]. Thus, there are different ratios of active and inactive TK1 in sera from dogs with different types of tumor disease as demonstrated in this study. Still, we find an overall correlation between the two assays both for patients with hematologic and solid tumors (*rs* = 0.77, P<0.0001 and *rs* = 0.36, P = 0.0098, respectively).

Here we also showed that TK1 protein levels returned to normal levels during chemotherapy of dogs with lymphoma, indicating disease remission. In two dogs with progressive lymphoma, the TK1 protein levels increased and these relapsed patients were euthanized. A similar pattern was found measuring the TK1 activity levels in these dogs, in accordance with earlier studies. A transient increase in TK1 protein and activity levels were found in a few patients during therapy, the reason of which is not currently known. The availability of the new ELISA should increase the clinical utility of TK1 as a proliferation biomarker and be valuable for prognosis and monitoring of dogs with hematological and solid tumors. However, larger multicenter clinical studies with larger number of dogs with different malignancies are needed to validate the role of TK1-ELISA in veterinary oncology.

## Conclusions

The present study describes a new TK1-ELISA for determining TK1 protein levels, which is a potential marker for diagnosis and monitoring of canine hematological tumors. The TK1-ELISA is easy to perform, fast, sensitive and as specific as the existing TK1 activity assays. The results presented with the new ELISA serve as the basis for a future development of a clinical immunoassay suitable for solid tumor diseases.

## Supporting Information

S1 File contains 3 tables: Table A, Table B and Table C that provide more information about each patient in each group. (DOCX)
