# Supplementary material for: A New Sandwich ELISA for Quantification of Thymidine Kinase 1 Protein Levels in Sera from Dogs with Different Malignancies Can Aid in Disease Management
Source: PLoS One. 2015 Sep 14;10(9):e0137871. doi: 10.1371/journal.pone.0137871 (PMC4569288; doi:10.1371/journal.pone.0137871)
Supplement: S1 File — (DOCX) [file pone.0137871.s001.docx]

**S1 File:**

**Table A: Thymidine kinase 1 (TK1) activity, and TK1 protein levels in sera from healthy dogs.**

|  |  |  |  | **STK1 activity** | **STK1-ELISA** |
| --- | --- | --- | --- | --- | --- |
| **S.No** | **Age** | **Sex** | **Breed** | **(pmol/min/mL)** | **(ng/mL)** |
|  | **(Years)** |  |  | **(Mean±SD)ͣ** | **(Mean±SD)ᵇ** |
|  |  |  |  |  |  |
| 1 | 8 | M | Labrador Retriever | 1.17±0.05 | 0.38±0.06 |
| 2 | 10 | F | Berner Sennenhund | 1.21±0.12 | 0.43±0.06 |
| 3 | 5 | F | Labrador Retriever | 0.98±0.07 | 0.33±0.03 |
| 4 | 6 | F | Golden Retriever | 0.93±0.06 | 0.30±0.04 |
| 5 | 5 | F | Golden Retriever | 0.96±0.05 | 0.20±0.03 |
| 6 | 6 | M | German Shepherd | 0.90±0.07 | 0.13±0.02 |
| 7 | 7 | M | Riesenschnauzer | 1.02±0.08 | 0.12±0.03 |
| 8 | 6 | F | Mixed Breed | 1.37±0.11 | 0.27±0.04 |
| 9 | 8 | F | Riesenschnauzer | 1.06±0.09 | 0.40±0.07 |
| 10 | 6 | M | German Shepherd | 0.90±0.07 | 0.24±0.05 |
| 11 | 5 | F | Rottweiler | 1.03±0.1 | 0.42±0.09 |
| 12 | 3 | M | English springer spaniel | 0.89±0.06 | 0.30±0.07 |
| 13 | 7 | F | Riesenschnauzer | 0.83±0.05 | 0.08±0.02 |
| 14 | 5 | M | Labrador Retriever | 1.04±0.08 | 0.24±0.07 |
| 15 | 6 | F | Mixed Breed | 0.73±0.06 | 0.1±0.02 |
| 16 | 8 | F | Golden Retriever | 0.76±0.07 | 0.22±0.05 |
| 17 | 7 | M | Riesenschnauzer | 1.01±0.09 | 0.22±0.04 |
| 18 | 7 | F | Flat coated Retriever | 1.12±0.1 | 0.11±0.03 |
| 19 | 6 | M | Schäfer | 1.10±0.14 | 0.47±0.08 |
| 20 | 6 | F | Riesenschnauzer | 1.13±0.09 | 0.35±0.07 |
| 21 | 5 | M | Labrador Retriever | 1.12±0.11 | 0.33±0.05 |
| 22 | 6 | F | Golden Retriever | 0.77±0.08 | 0.22±0.02 |
| 23 | 7 | F | Labrador Retriever | 1.02±0.09 | 0.18±0.03 |
| 24 | 7 | M | Labrador Retriever | 1.08±0.08 | 0.22±0.06 |
| 25 | 4 | M | Labrador Retriever | 1.17±0.12 | 0.32±0.05 |
| 26 | 7 | M | Schäfer | 1.28±0.17 | 0.35±0.06 |
| 27 | 7 | M | Leonberger | 0.83±0.11 | 0.24±0.06 |
| 28 | 3 | M | Schäfer | 1.31±0.16 | 0.36±0.07 |
| 29 | 6 | M | Golden Retriever | 0.89±0.14 | 0.07±0.02 |
| 30 | 5 | M | Flat coated Retriever | 0.96±0.09 | 0.42±0.09 |
| 31 | 2 | M | Labrador retriever | 1.79±0.12 | 0.37±0.07 |
| 32 | 6 | M | Berense Mountian Dog | 1.43±0.14 | 0.27±0.06 |
| 33 | 2 | F | Berense Mountian Dog | 1.82±0.13 | 0.37±0.06 |
| 34 | 5 | M | Mixed breed | 1.38±±0.09 | 0.15±0.02 |
|  |  |  |  |  |  |
| 35  36 | 2  7 | M  M | German Shepherd  Golden Retriever | 1.59±0.11  1.43±0.09 | 0.34±0.04  0.31±0.05 |
| 37 | 7 | M | Mixed breed | 1.31±0.1 | 0.26±0.03 |
| 38 | 3 | M | Boxer | 1.09±0.07 | 0.23±0.02 |
| 39 | 3 | M | Mixed breed | 1.12±0.06 | 0.24±0.02 |
| 40 | 2 | F | Golden Retriever | 0.97±0.07 | 0.20±0.03 |
| 41 | 3 | M | Mixed breed | 1.21±0.11 | 0.28±0.04 |
| 42 | 5 | F | Labrador Retriever | 1.32±0.09 | 0.32±0.06 |

ͣ Mean values of three observations from a single experiment. ᵇ Mean values of two observations from two independent experiments.

**Table B:** **Thymidine kinase 1 activity and protein levels in sera from dogs with hematological malignancies.**

|  |  |  |  |  | **STK1 activity** | **STK1 protein** |
| --- | --- | --- | --- | --- | --- | --- |
| **S.No** | **Age** | **Sex** | **Breed** | **Diagnosis** | **(pmol/min/mL)** | **(ng/mL)** |
|  | **(Years)** |  |  |  | **(Mean±SD)ͣ** | **(Mean±SD)ᵇ** |
|  |  |  |  |  |  |  |
| 1 | 13 | F | Bull terrier | Lymphoma | 8.52±1.26 | 0.56±0.04 |
| 2 | 8 | M | Riesen Schnauzer | Lymphoma | 2.65±0.34 | 3.64±0.72 |
| 3 | 7 | F | Mixed Breed | Lymphoma | 17.1±2.12 | 1.86±0.43 |
| 4 | 6 | M | Riesen Schnauzer | Lymphoma | 2.87±0.54 | 0.64±0.03 |
| 5 | 8 | M | Rottweiler | Lymphoma | 4.72±0.58 | 0.87±0.12 |
| 6 | 8 | F | Golden Retriever | Leukemia | 38.3±4.32 | 4.19±.0.46 |
| 7 | 6 | F | Nova Scotia Retriever | Lymphoma | 2.43±0.14 | 0.52±0.05 |
| 8 | 9 | F | Bernersennenhund | Lymphoma | 13.1±2.26 | 1.71±0.18 |
| 9 | 6 | F | Nova Scotia Retriever | Lymphoma | 7.23±2.45 | 1.31±0.17 |
| 10 | 10 | M | Doberman | Lymphoma | 1.95±0,06 | 0.25±0.03 |
| 11 | 13 | F | Clumber Spaniel | Lymphoma | 1.89±0.08 | 0.76±0.10 |
| 12 | 10 | F | Fox terrier | Lymphoma | 0.54±0.04 | 0.39±0.06 |
| 13 | 12 | F | West highland white terrier | Lymphoma | 1.88±0.14 | 0.45±0.06 |
| 14 | 11 | M | Shetland sheep dog | Leukemia | 1.33±0.07 | 0.33±0.05 |
| 15 | 12 | F | Riesen Schnauzer | Lymphoma | 1.61±0.09 | 0.56±0.07 |
| 16 | 6 | F | Nova Scotia Retriever | Lymphoma | 0.75±0,04 | 0.36±0.05 |
| 17 | 8 | F | Boxer | Lymphoma | 6.16±1.04 | 0.71±0.11 |
| 18 | 10 | F | Labrador Retriever | Lymphoma | 13.6±2.16 | 1.87±0.36 |
| 19 | 9 | F | Scottish Terrier | Lymphoma | 14.4±2.67 | 1.76±0.31 |
| 20 | 6 | F | Gordon Setter | Lymphoma | 0.83±0.05 | 0.35±0.05 |
| 21 | 9 | F | Rottweiler | Leukemia | 58.7±6.38 | 4.07±0.58 |
| 22 | 9 | F | Briard | Lymphoma | 0.86±0.06 | 0.41±0.07 |
| 23 | 7 | M | Golden Retriever | Lymphoma | 22.8±2.13 | 2.48±0.26 |
| 24 | 8 | F | Hovawart | Lymphoma | 14.4±1.31 | 4.38±0.54 |
| 25 | 8 | M | Rottweiler | Lymphoma | 5.91±1.84 | 0.87±0.30 |
| 26 | 11 | M | Norwich Terrier | Lymphoma | 1.28±0.08 | 0.95±0.19 |
| 27 | 7 | F | Mixed Breed | Lymphoma | 2.17±0,12 | 0.95±0.16 |
| 28 | 10 | F | Rottweiler | Lymphoma | 1.56±0.11 | 0.67±0.11 |
| 29 | 8 | F | Fox terrier | Lymphoma | 1.02±0.06 | 0.39±0.05 |
| 30 | 9 | F | Norwich Terrier | Lymphoma | 4.68±1.64 | 1.95±0.33 |
| 31 | 10 | M | Golden Retriever | Leukemia | 39.3±4.48 | 3.92±0.78 |
| 32 | 8 | M | Labrador Retriever | Leukemia | 5.72±1.78 | 1.32±0.22 |
| 33 | 9 | F | Mixed Breed | Lymphoma | 29.8±5.23 | 2.48±0.41 |
| 34 | 7 | M | Mixed Breed | Lymphoma | 1.62±0.07 | 1.50±0.28 |
|  |  |  |  |  |  |  |
| 35 | 3 | F | Swedish Elkhound | Lymphoma | 1.09±0.1 | 0.78±0.21 |
| 36 | 10 | F | Labrador Retriever | Lymphoma | 0.61±0.05 | 0.53±0.07 |
| 37 | 6 | F | Rottweiler | Lymphoma | 3.54±0.33 | 0.70±0.09 |
| 38 | 11 | F | Curly coated Retriever | Lymphoma | 1.5±0.05 | 0.39±0.04 |
| 39 | 9 | F | Briard | Lymphoma | 11.9±0.98 | 0.93±0.10 |
| 40 | 9 | M | Rhodesian ridge back | Lymphoma | 2.14±0.23 | 0.48±0.05 |
| 41 | 6 | F | Finnish Hound | Lymphoma | 2.66±0.34 | 0.71±0.09 |
| 42 | 4 | M | Rhodesian ridge back | Lymphoma | 6.32±0.58 | 0.51±0.04 |
| 43 | 7 | M | Whippet | Lymphoma | 9.18±1.12 | 1.18±0.14 |

ͣ Mean values of three observations from a single experiment. ᵇ Mean values of two observations from two independent experiments.

**Table C:** **Thymidine kinase 1 activity and TK1-ELISA protein levels sera from dogs with solid tumors**

|  |  |  |  |  | **STK1 activity** | **STK1-ELISA** |
| --- | --- | --- | --- | --- | --- | --- |
| **S.No** | **Age** | **Sex** | **Breed** | **Diagnosis** | **(pmol/min/mL)** | **(ng/mL)** |
|  | **(Years)** |  |  |  | **(Mean±SD)ͣ** | **(Mean±SD)ᵇ** |
|  |  |  |  |  |  |  |
| 1 | 6 | F | Rottweiler | Mammary Carcinoma | 0.72±0.05 | 0.28±0.03 |
| 2 | 10 | F | Field Spaniel | Mammary Adenoma | 0.81±0.07 | 0.46±0.08 |
| 3 | 8 | F | Golden Retriver | Mammary Carcinoma | 1.51±0.12 | 1.22±0.23 |
| 4 | 6 | F | Rottweiler | Mammary Adenoma | 0.87±0.04 | 0.40±0.03 |
| 5 | 3 | F | Rhodesian ridgeback | Mammary Adenoma | 0.62±0.04 | 0.46±0.12 |
| 6 | 10 | F | English Springer Spaniel | Mammary Adenoma | 1.52±0.07 | 0.43±0.09 |
| 7 | 11 | F | Rhodesian ridgeback | Mammary Adenoma | 0.94±0.09 | 0.56±0.07 |
| 8 | 14 | F | Brooder Coolie | Mammary Carcinoma | 1.61±0.12 | 0.77±0.18 |
| 9 | 13 | F | Boxer | Mammary Carcinoma | 0.93±0.07 | 0.47±0.09 |
| 10 | 6 | F | Mixed breed | Mammary Carcinoma | 1.12±0.09 | 0.73±0.13 |
| 11 | 11 | F | Boxer | Mammary Adenoma | 1.43±0.14 | 0.97±0.10 |
| 12 | 6 | F | Flat Coated Retriever | Mammary Adenoma | 1.02±0.07 | 0.77±0.08 |
| 13 | 9 | F | Rottweiler | Mammary Adenoma | 1.32±0.09 | 1.50±0.21 |
| 14 | 12 | F | Grey Hound | N.D | 1.83±0.12 | 0.47±0.08 |
| 15 | 10 | F | German Shepherd | Mammary Carcinoma | 1.31±0.07 | 0.54±0.07 |
| 16 | 7 | F | English Bull dog | Mammary Carcinoma | 25.8±2.9 | 2.22±0.24 |
| 17 | 6 | F | English Springer Spaniel | Mammary Adenoma | 2.02±0.20 | 0.94±0.11 |
| 18 | 10 | F | English springer spaniel | Mammary Carcinoma | 1.54±0.11 | 0.84±0.16 |
| 19 | 9 | F | Welsh springer spaniel | Mammary Adenoma | 2.62±0.22 | 0.21±0.05 |
| 20 | 11 | F | Cairn terrier | N.D | 1.39±0.08 | 0.35±0.09 |
| 21 | 5 | F | Miniature poodle | Mammary Carcinoma | 2.25±0.18 | 0.48±0.08 |
| 22 | 12 | F | Schäfer | Mammary Adenoma | 0.81±0.11 | 0.22±0.04 |
| 23 | 8 | M | Blandras | Histyocytic Sarcoma | 1.08±0.08 | 0.34±0.05 |
| 24 | 7 | M | Cocker Spaniel | Mastocytoma (II) | 1.46±0.09 | 0.25±0.07 |
| 25 | 8 | M | French Bull dog | Malignant Melanoma | 0.88±0.09 | 0.21±0.06 |
| 26 | 6 | F | Rottweiler | Malignant Melanoma | 1.03±0.14 | 0.27±0.07 |
| 27 | 8 | F | Labrador Retriever | HemangioSarcoma | 1.78±0.18 | 1.13±0.15 |
| 28 | 7 | F | Flat Coated Retriever | HemangioSarcoma | 0.53±0.05 | 0.35±0.08 |
| 29 | 9 | F | Riesen Schnauzer | Malignant Melanoma | 1.99±0.17 | 2.10±0.37 |
| 30 | 8 | F | Blandras | Mastocytoma (III) | 0.51±0.04 | 0.66±0.11 |
| 31 | 8 | F | Weimarner | Mastocytoma (II) | 0.67±0.06 | 0.53±0.04 |
| 32 | 6 | F | Labrador Retriever | Mastocytoma (II) | 1.04±0.11 | 0.48±0.07 |
| 33 | 10 | F | Pug | Mastocytoma (II) | 0.53±0.06 | 0.42±0.09 |
| 34 | 10 | F | Labrador Retriever | Mastocytoma (III) | 1.52±0.11 | 1.11±0.16 |
| 35 | 7 | F | Blandras | Mastocytoma (II) | 1.08±0.08 | 0.61±0.11 |
| 36 | 14 | M | Golden Retriever | Malignant Melanoma | 1.19±0.14 | 0.27±0.05 |
|  |  |  |  |  |  |  |
| 37 | 13 | M | Blandras | Malignant Melanoma | 1.26±0.11 | 0.34±0.09 |
| 38 | 12 | M | Golden Retriever | Malignant Melanoma | 1.48±0.14 | 0.68±0.12 |
| 39 | 9 | M | Rottweiler | Malignant Melanoma | 1.01±0.09 | 0.92±0.21 |
| 40 | 6 | M | Schnauzer | Malignant Melanoma | 0.94±0.08 | 0.53±0.1 |
| 41 | 14 | M | Golden Retriever | Malignant Melanoma | 2.42±0.19 | 3.40±0.48 |
| 42 | 7 | M | Flat Coated Retriever | Histyocytic Sarcoma | 1.28±0.09 | 0.67±0.17 |
| 43 | 7 | M | Rottweiler | Malignant Melanoma | 1.15±0.13 | 0.62±0.09 |
| 44 | 9 | M | Dvårgschnauzer | Malignant Melanoma | 0.52±0.06 | 0.51±0.08 |
| 45 | 3 | F | Australian Shepherd | AdenoCarcinoma | 0.58±0.07 | 0.72±0.11 |
| 46 | 7 | M | Staffordshire bull terrier | Mastocytoma (I) | 0.61±0.08 | 0.36±0.07 |
| 47 | 11 | M | Mixed breed | Malignant Melanoma | 1.51±0.21 | 0.45±0.12 |
| 48 | 10 | M | Labrador Retriever | Squamous cell Carcinoma | 1.29±0.12 | 0.38±0.08 |
| 49 | 5 | M | Labrador Retriever | Nasal Tumor | 3.36±0.34 | 0.65±0.13 |
| 50 | 10 | F | Lagotto Romagnolo | Squamous cell Carcinoma | 1.89±0.19 | 0.48±0.09 |
| 51 | 9 | M | Cavalier charles spaniel | Squamous cell Carcinoma | 1.25±0.15 | 0.22±0.05 |
| 52 | 6 | M | Nova scotia duck Retriever | Thyroid CarcinoSarcoma | 1.84±0.14 | 0.41±0.08 |
| 53 | 7 | F | Labrador Retriever | Mastocytoma (II) | 1.25±0.11 | 0.47±0.09 |
| 54 | 5 | M | Rhodesian ridgeback | Mastocytoma (II) | 1.21±0.12 | 0.55±0.1 |
| 55 | 1 | F | Labrador Retriever | Infilterative Lipoma | 1.61±0.09 | 0.64±0.12 |

ͣ Mean values of three observations from a single experiment. ᵇ Mean values of two observations from two independent experiments. Mastocytomas are graded G-I, II, and III. Mammary tumors are classified as benign (adenomas), malignant (carcinomas) and not differentiated (ND).
